# Knee Flexion While Walking Exceeds Knee Flexion Contracture in Children with Spastic Cerebral Palsy

**DOI:** 10.3390/children10121867

**Published:** 2023-11-29

**Authors:** Åsa Bartonek, Cecilia Lidbeck

**Affiliations:** 1 Division of Paediatric Neurology, Department of Women’s and Children’s Health, Karolinska Institutet, SE-17176 Stockholm, Sweden; cecilia.lidbeck@ki.se; 2 Motion Analysis Laboratory, Karolinska University Hospital, QA:27, Karolinska vägen 37A, SE-17176 Stockholm, Sweden

**Keywords:** gait analysis, mobility, sensorimotor, posture

## Abstract

Flexed knee gait is commonly related to contractures in children with cerebral palsy (CP). Therefore, knee position while walking was compared with passive knee extension and explored with respect to functional mobility. Gait was assessed with 3D motion analysis in 30 children with bilateral spastic CP, Gross Motor Function Classification System (GMFCS) levels I–III, and in 22 typically developing (TD) children. Knee angle at initial contact (KneeAngleIC) was greater than knee flexion in stance (MinKneeFlexSt) in all groups. MinKneeFlexSt exceeded knee contractures at GMFCS levels II and III. Both KneeAngleIC and MinKneeFlexSt were greater at GMFCS II and III than at GMFCS I and the TD group. The excessive knee flexion while walking at GMFCS II and III could not be explained by knee joint contractures. Functional mobility measured with the timed-up-and-go test took longer in children at GMFCS level III compared to the other groups, assumed to be explained by the energy-requiring flexed knee gait and spatial insecurity. Discriminating between passive knee extension at the physical assessment and maximum knee extension while weight bearing may contribute to further understanding of flexed knee gait and its causes in ambulating children with spastic bilateral CP.

## 1. Introduction

Cerebral palsy (CP) is a term covering a group of permanent but not unchanging disorders of movement and/or posture that causes activity limitation in the developing brain [1]. Motor functioning is most commonly graded according to the Gross Motor Function Classification System (GMFCS) [2]. The motor disorders often go together with, e.g., disturbances of sensation and perception and with musculoskeletal problems [1]. In high-income countries, the birth prevalence of CP is estimated at 1.6 per 1000 live births, markedly higher than in low- and middle-income countries [3]. There are updated guidelines for improvement of physical function for children and young people with CP [4]. Regarding walking ability, including walking speed and physical endurance, overground walking with or without a walker is recommended for GMFCS levels I–IV and treadmill training for GMFCS levels I–III [4].

As gait pattern matures in children with CP, the development of flexed knee gait is frequently seen. In particular in children with diplegia, short hamstring muscles may occur; if not treated, this leads to a knee flexion contracture. For ambulatory individuals, limited ability for full knee extension may lead to significant disability with a flexed knee gait posture, called crouch gait [5,6]. The impact of this musculoskeletal deformity has been found to be a significant factor in maintaining or losing walking ability at age 16 in children with CP [7]. Recently, knee contracture was reported to occur in 22% in a group with a verified CP diagnosis in a register-based study of more than 3000 children. The contracture was described as mild in 12% and as severe in 10% of the children. The prevalence was found to be higher in older children, with increasing GMFCS level and in the presence of short hamstring muscles, whereby spasticity was found to have a small effect [8].

As early as the 1970s, Sutherland and Cooper [9] recommended gait analysis techniques to capture excessive stance-phase knee flexion during walking in children with CP. In the 1990s, the definition of crouch knee gait as a knee flexion of at least 30 degrees throughout the stance phase was introduced [10]. By linking gait patterns to intervention strategies concerning spasticity, muscle contracture lengthening, and the choice of orthotics, Rodda et al. [11] proposed a classification of gait in five patterns, including crouch gait. To allow an even more objective identification of gait patterns in the sagittal plane, a computational classification of a plantarflexor-knee-extension couple index was suggested to be useful in patients with CP in clinical trials [12,13]. More recently, the various gait patterns in CP were suggested to be a continuum of various deviations rather than well-delineated groups. To distinguish primary deviations from compensatory kinematic strategies during walking, the importance of linking impairments from results of the clinical examination to gait deviations was highlighted [14]. Pain, on the other hand, was not found to be associated with the amount of knee flexion in stance but rather with the presence of patella alta [15]. In a study exploring long-term outcomes after orthopedic surgery to correct flexed knee gait in children with spastic diplegia, the authors concluded that possible overcorrection should be considered in treatment planning [16]. According to a systematic literature review on interventions to improve crouch gait patterns in CP, identification of interventions to improve sagittal plane knee motion largely failed, with limited evidence of improved gait speed or motor function [17]. Recently, a new system to trigger electrical stimulation of the quadriceps muscles in a pediatric robotic exoskeleton was tested, resulting in an immediate improvement to knee extension during stance; this is suggested to be investigated further as a treatment for children with crouch gait [18].

Flexed knee gait has been described as a complex multidimensional deformity in children whose natural history is variable [5]. Moreover, the energy-demanding walking patterns of crouch gait have been considered to have an underlying cause beyond entirely biomechanical issues, suggesting that generally recognized principles of crouch gait and its treatment are insufficiently understood and need to be further investigated [15]. When debating the causes of flexed knee gait in ambulant children with spastic CP, the central organization of posture has been mentioned less often than biomechanical factors. Besides body posture being connected by flexible joints and controlled by the neuromuscular system, the central organization of posture involves multisensory inputs from vision, balance organs, proprioception, and skin, which contribute to postural orientation with respect both to one another and to the vertical gravity vector [19]. In fact, CP has been suggested to be a sensorimotor disorder [20]; however, the vocabulary used to describe and categorize impairments still stresses motor rather than sensorimotor characteristics. Moreover, among the forms of spastic diplegia, children have been characterized as mainly being influenced by motor disturbances, whereas other children have been described as presenting with perceptual disorders causing fear and uneasiness when moving [21]. To distinguish gait in these various clinical forms of spastic diplegia, the central components of motor organization were collected by means of an optoelectronic system that automatically extracts specific angular parameters [22]. In a research project comprising a cohort of children with bilateral spastic CP (BSCP), standing [23] was investigated with three-dimensional motion analysis. While walking during a demanding task of turning [24], significant deviations in kinematic as well as in time and distance parameters were found in children at GMFCS levels II and III compared to those at level I and children with typical development (TD). In addition, while performing activities of crawling and kneeling, children at GMFCS level III had reduced ability in standing and walking compared to children at the other levels [25]. The findings of these latter studies referred not only to the child’s motor disorder but also to disturbances in lower limb proprioception and difficulties with spatial orientation. In one of the above-mentioned studies, children with BSCP had difficulties in maintaining knee extension against gravity while standing [23], which was not considered to be associated with knee extensor muscle weakness. This difficulty was confirmed in a study assessing knee joint position sense in sitting in children with various groups of motor disabilities with good quadriceps muscle strength, grade 4–5, on a six-grade scale [26]. In this study, however, it turned out that the CP group had greater difficulties than the other groups in maintaining the knee position during the trial; a result that points to the need for complementary judgments of joint proprioception and possible consequences on knee position during walking [27]. Based on the research findings in children with BSCP, seen as difficulties in maintaining a knee position that is more extended while standing than their possible passive knee extension [23], the aim of the present study was to compare knee position during walking with passive knee extension in an unloaded body position. The hypothesis was that knee flexion while walking would exceed that of the knee flexion contracture. A secondary aim was to explore the relationship between knee flexion during gait and the child’s functional walking.

## 2. Materials and Methods

### 2.1. Participants

In this observational cross-sectional study on a dataset of subjects with BSCP, who were examined between January 2012 and September 2013 in the Motion Analysis Laboratory at Karolinska University Hospital Stockholm, gait was retrospectively analyzed. There were 30 participants, 13 females and 17 males, with a median (min, max) age of 11.3 (7.6, 17.1) years. Inclusion criteria were GMFCS levels I–III [2]. Exclusion criteria were botulinum toxin treatment and soft tissue orthopedic surgery within the past six months, and bony surgery within the past year. Twenty-two children with typical development (TD), 11 females and 11 males, with a median (min-max) age of 8.9 (6.5–16.9), comprised a control group (TD). The number of orthopedic lower limb surgeries that had been performed was obtained from the medical chart.

The study was conducted in accordance with the Declaration of Helsinki and approved by the Regional Ethics Board in Stockholm Sweden (No. 2011/596-31/1, No. 2012/2054-32). Written consent was obtained from the parents of the participating children.

### 2.2. Joint Contractures

Contractures in the lower limb joints, defined from a neutral position, were assessed through goniometric measurement, which is a valid method for measurement of joint range of motion [28]. Knee extension and ankle dorsiflexion with extended knee were measured in the supine position [29]. Hip flexion contracture was measured using the method specified by Bartlett [30].

### 2.3. Movement Analysis

Three-dimensional motion analysis using eight cameras (Vicon, Oxford, UK), with a full-body marker set (Plug-In-Gait), was used for kinematic analyses [31]. The children walked barefoot on an 8 m walkway with self-selected walking speed. Knee position was calculated at initial foot contact (KneeAngleIC) and at minimum knee flexion in stance (MinKneeFlexSt). The most supporting leg was chosen for analysis, as determined from force plate data (Kistler^®^, Winterthur, Switzerland) during standing. The first kinematic trial with complete marker data was analyzed.

### 2.4. Functional Mobility

The Timed Up and Go test (TUG) [32] was used to assess the child’s functional mobility while walking. The children were instructed to stand up, walk three meters around a target, return, and sit down again. Timing was started as the participant left the seat and stopped as their bottom touched the chair.

### 2.5. Statistics

Descriptive data were presented as median, minimum, and maximum values. The Kruskal–Wallis test and the Mann–Whitney *U* test with post hoc adjustments were used to analyze KneeAngleIC and MinKneeFlexSt values, and the time to complete the TUG test with respect to the different GMFCS levels and the TD group. The Wilcoxon signed-rank test was used to compare knee angles within GMFCS groups and the TD group. Spearman rank correlation coefficients were used to examine associations between TUG and knee angles at initial contact and in stance. A correlation coefficient (r_s_) of 0.00–0.20 was interpreted as slight correlation, 0.21–0.40 as fair, 0.41–0.60 as moderate, 0.61–0.80 as substantial, and 0.81–1.00 as almost perfect [33]. A chi-squared test was used for the analysis of orthopedic surgeries performed with respect to the groups. Statistical significance was set at *p* < 0.05, and calculations were performed using the statistical program SPSS 26.0.

## 3. Results

There were no differences in age, weight, or height with respect to GMFCS groups and the TD group. Orthopedic surgery in the lower limbs had been performed in 10 out of 30 children with CP. No difference was found between the groups in number of performed orthopedic surgeries (Table 1).

### 3.1. Joint Contractures

Ankle plantarflexion and hip flexion contractures did not differ between the groups. The post hoc Mann–Whitney *U* test revealed that knee contractures were greater in the GMFCS III group than the neutral joint position that was present in GMFCS I and the TD group (*p* = 0.046 and *p* = 0.002, respectively) (Table 1).

### 3.2. Knee Position at Initial Foot Contact (KneeAngleIC)

KneeAngleIC differed significantly between the TD and the GMFCS groups (Table 2). The post hoc Mann–Whitney *U* test showed that KneeAngleIC was greater in the GMFCS II and III groups than in the TD group (*p* < 0.001, *p* < 0.001), greater in GMFCS II and III than in GMFCS I (*p* = 0.006 and *p* = 0.003, respectively), and greater in GMFCS III than in GMFCS II (*p*= 0.046).

### 3.3. Minimum Knee Flexion in Stance (MinKneeFlexSt)

MinKneeFlexSt differed significantly between the TD and the GMFCS groups (Table 2). The post hoc Mann–Whitney *U* test showed that MinKneeFlexSt was greater in GMFCS II and III than in TD (*p* < 0.001 and *p* < 0.001, respectively), and greater in GMFCS II and GMFCS III than in GMFCS I (*p* = 0.048 and *p* = 0.019, respectively).

**Figure 1:**
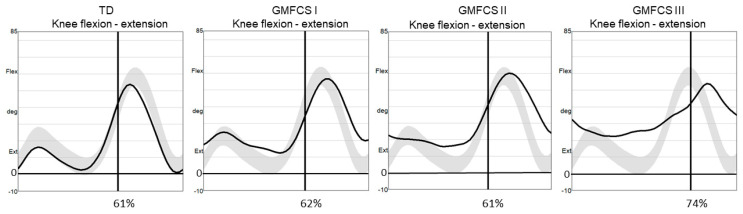
Kinematic illustration of knee flexion as a mean of three trials during a gait cycle in a TD child (typically developing) and a child with cerebral palsy at GMFCS I, GMFCS II, and GMFCS III (two trials). Percentage of stance phase is indicated on the vertical line. The shaded field represents the mean ± 1 standard deviation of the gait laboratory control group.

Figure 1 exemplifies knee kinematics during a gait cycle on the support leg in a TD child and in one individual in GMFCS I, II, and III groups.

### 3.4. Knee Contractures versus Minimum Knee Flexion in Stance (MinKneeFlexSt)

**Figure 2:**
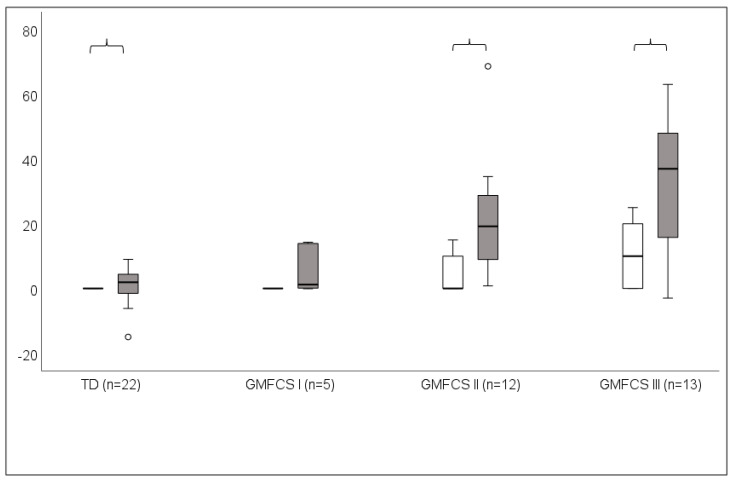
Knee contractures and MinKneeFlexSt (minimum knee flexion angle in stance) presented as median and 25th–75th percentiles in TD group (children with typical development) and children with bilateral spastic cerebral palsy (BSCP), GMFCS levels I–III. White bars represent passive knee extension, grey bars represent MinKneeFlexSt. (+) indicates knee flexion. Brackets denote statistically significant difference (*p* < 0.05).

Knee contractures were smaller than MinKneeFlexSt in TD (*p =* 0.046), GMFCS II (*p* = 0.004), and GMFCS III (*p* = 0.002) but not in GMFCS I (*p =* 0.104) (Figure 2).

### 3.5. Knee Position at Initial Foot Contact (KneeAngleIC) versus Minimum Knee Flexion in Stance (MinKneeFlexSt)

**Figure 3:**
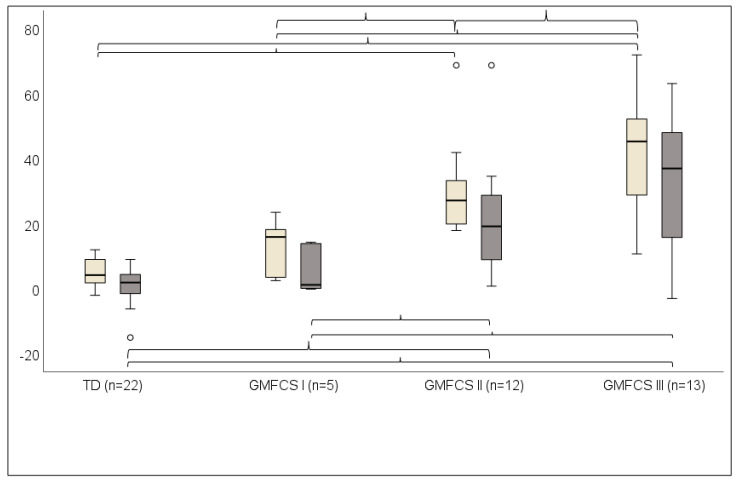
KneeAngleIC (knee angle at initial contact) and MinKneeFlexSt (minimum knee flexion angle in stance) presented as median and 25th–75th percentiles in TD group (children with typical development) and children with bilateral spastic cerebral palsy (BSCP), GMFCS levels I–III. Light grey bars represent KneeAngleIC, and dark grey bars represent MinKneeFlexSt. (+) indicates knee flexion. Brackets indicate significant difference (*p* < 0.05). Brackets above the boxes show group differences at KneeAngleIC, and brackets below the boxes show group differences at MinKneeFlexSt.

KneeAngleIC was significantly greater than MinKneeFlexSt in TD (*p =* 0.008), GMFCS I (*p =* 0.043), GMFCS II (*p =* 0.005), and GMFCS III (*p =* 0.002) (Figure 3).

### 3.6. Timed up and Go Test (TUG)

Time to perform the TUG test (measured in seconds) differed significantly between the GMFCS levels and the TD group (Table 2). The post hoc Mann–Whitney *U* test revealed significantly longer times for GMFCS II and GMFCS III compared to TD (*p* < 0.001 and *p* < 0.001, respectively) and GMFCS I (*p =* 0.001 and *p* < 0.001, respectively). Times were also longer for GMFCS III compared to GMFCS II (*p* < 0.001).

**Figure 4:**
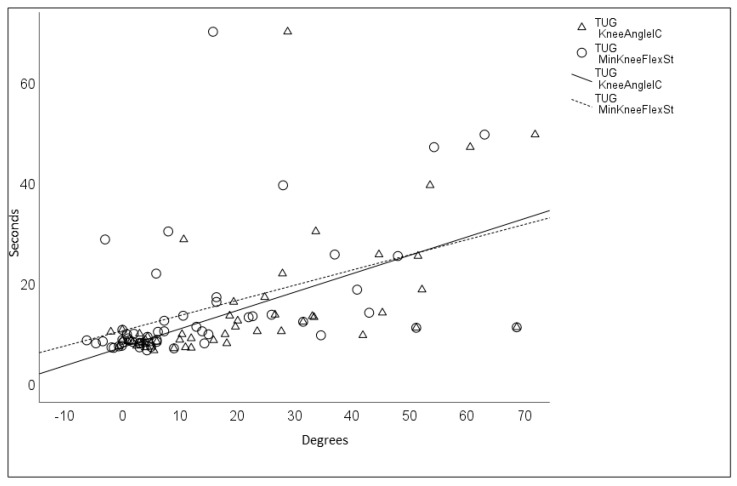
Correlation between TUG (Timed Up and Go test) and KneeAngleIC (knee angle at initial contact) and between TUG and MinKneeFlexSt (minimum knee flexion angle in stance), expressed as Spearman’s rank correlation coefficient values in groups of typically developing children and those with bilateral spastic cerebral palsy.

Between the TD group and the entire BSCP group, the Spearman’s rank correlation coefficient was almost perfect between TUG and KneeAngleIC (0.843, *p* < 0.001) and was substantial between TUG and MinKneeFlexSt (0.683, *p* < 0.001). These results indicate that the more time taken to complete the TUG test, the greater the knee flexion angles are during walking (Figure 4).

## 4. Discussion

In this study, the main purpose was to examine knee position during walking with respect to knee contractures in children with bilateral spastic CP. In agreement with our hypothesis, we found that knee flexion during walking was greater than knee contractures measured as passive knee extension; it was significantly greater in children at GMFCS levels II and III but not at GMFCS level I. In children at GMFCS I, the possible passive knee extension was utilized in stance, whereas the children at GMFCS II and III walked with considerably greater knee flexion angles in stance compared to knee contractures. In both the GMFCS II and III groups, there seemed to be an inability to utilize the available knee flexion angle during walking of approximately 20 degrees at GMFCS level II and 40 degrees at GMFCS level III compared to the TD group, who presented with a difference of approximately four degrees. The knee contractures measured in the present study were greater at GMFCS levels II and III than at level I. The result seems consistent with those presented in the study of Cloodt [8], wherein knee contractures increased from GMFCS I to GMFCS III. Similarly, they also found a large heterogeneity within the GMFCS levels, with knee extension ranging from normal, above −4 degrees, to below −15 degrees, which was defined as severe contracture. Since CP subtypes were not differentiated in the latter study, no further considerations with respect to the present study group can be made.

In addition to minimum knee flexion in stance, we analyzed the knee flexion angle at initial foot contact. Both the latter angle and that of knee flexion in stance were greater in GMFCS II and III than those in the TD group, and increasingly greater with the more severe motor function difficulties at higher GMFCS levels, except in stance at GMFCS I, than in the TD group. This last finding may be in agreement with Sutherland and Cooper [9], who reported that children with CP who show some increase in knee flexion at foot contact compared to TD, followed by full knee extension in stance, were without risk of developing a crouch gait. In our study, we found that children at GMFCS II walked with a knee flexion angle of almost 30 degrees at foot contact and 20 degrees in stance, whereas in GMFCS III, the corresponding angles were almost 50 and 40 degrees, respectively. In the study of Sutherland and Davids [10], participants with increased knee flexion of at least 30 degrees throughout the stance phase were defined as having crouch knee gait. In the present study, only children at GMFCS level III fit this definition, even though some children at GMFCS level II could also be designated as having a crouched gait. Although at various degrees, the same walking pattern with larger knee angles at initial foot contact than in stance could be confirmed at all GMFCS levels and in the TD group.

In the study of Sutherland and Cooper [9], all participants had gone through surgical Achilles tendon lengthening, which was assumed to have caused the crouched position due to calf muscle weakness. They suggested surgical release of the hamstring muscle to avoid knee flexion contractures and restore the strength of the quadriceps muscles. In our study group, a hamstring release had been performed in one limb only in a child functioning at GMFCS II. Strayer surgery or Achilles’s tendon lengthening had been performed in forty percent of the limbs, in GMFC I in 20% (1 of 5 children), in GMFCS II in 67% (8 of 12 children), and in GMFCS III in 15% (2 of 13 children),and did not reach significant levels between the groups. Moreover, Sutherland and Cooper [9] suggested that an additional increase in ankle dorsiflexion during stance may lead to pes valgus or external tibia torsion contributing to the increase in knee flexion during walking. In our study, passive ankle dorsiflexion did not vary between the children at the various GMFCS levels and did not exceed zero degrees in any child. However, as crouch gait often involves dorsiflexion of the foot in stance and foot postural problems [5], it is possible that instability in the foot and ankle could have influenced knee extension during weight bearing in our study. Using ankle-foot orthoses to stabilize the ankle during gait trials could have added valuable information about knee kinematics; however, not all participants were orthosis users, impeding a comparison being made between the children. Comparison of kinetic information that could have been of interest for evaluating joint moments during gait was also not possible, since children at GMFCS level III used walking aids that interfered with obtaining valid force plate data. However, despite the use of assistive devices enabling decreased loading on the legs, the children at GMFCS level III displayed the largest knee angles during walking of all groups in our study.

The increasing time to perform the TUG test [32] with respect to gradually increasing knee flexion angles was confirmed by the substantial to almost perfect correlations with knee position at initial contact and in stance, respectively. From a functional perspective, children at GMFCS levels II and III required significantly longer times than those in GMFCS I and TD groups to carry out the TUG test. This is in agreement with Williams et al. [32], who reported that children functioning at GMFCS level I performed the test significantly faster compared to children at levels II and III, as well as level II compared to III. The large difference found in our study can likely be explained by the energy-requiring flexed knee gait of the GMFCS III group, walking with twice as much knee flexion in stance as GMFCS II. Moreover, since the TUG test includes rising from the chair, walking a short distance, and returning to sitting, it is probable that the effort to overcome motor disorders such as spasticity determined mobility. Concerning spasticity, no relationship was found with respect to knee contracture in the plantar flexors, while reduced length of hamstring and gastrocnemius muscles presented an increased risk for knee contracture [8]. Therefore, spatial insecurity could have been another plausible cause for the long time needed to perform the TUG test in the children in the GMFCS III group, since the task included complex movements such as changing of position from sitting to standing and changing direction. Spatial insecurity has been suggested previously as a reason for low walking speed in children at GMFCS levels II and III during turning [24].

Cerebral palsy is by definition a disturbance in movement and posture [1]. The vocabulary commonly used today to describe movement and posture in children with CP may obscure the importance of the role of the sensory system [20]. A complementary view, therefore, could be that CP involves a lack of multisensory inputs necessary for postural orientation in relation to gravity [19]. The important question regarding whether it is sufficient to refer to flexed knee gait in children with CP in biomechanical terms [15] is illustrated in a participant from the GMFCS II group in this study. According to Rodda et al. [12], half of the children in their study had not undergone calf muscle surgery, suggesting that crouch gait in these children was part of the natural history of their gait disorder. This seems in line with the herein presented boy who had no orthopedic surgery of the legs nor had accepted ankle stabilizing orthoses at any time in childhood. Walking independently, he presented with more than sixty-degree flexion angles at foot contact as well as in stance, at no more than fifteen degrees of knee contracture. The boy’s parents reported that he had unstable trunk posture in sitting and was continually being told to straighten himself. Such difficulty in maintaining posture, having repeatedly to restore an upright sitting position, or being told by the caregiver to sit erect has been reported in children with spastic CP [21]. Similar behavior, such as difficulty in maintaining extension of the legs after rising to standing [34] may contribute to difficulties in orienting the postural segments [19]. There are varying recommendations regarding the improvement of walking ability, such as overground walking with or without a walker, treadmill training [4], and suggested electrical stimulation to the quadriceps muscles in a pediatric robotic exoskeleton [18]. Adding aspects of sensorimotor conditions to the motor performance may add valuable information on the choices of mobility among children at various GMFCS levels. It is worth mentioning the similar motor function in the C dimension in crawling and kneeling tasks of the gross motor function measurement that was found in the GMFCS III group as compared to levels I and II [2]. Since high-demanding postural tasks such as standing and walking were found to have significantly lower scoring in GMFCS III, there may be a risk of a prolonged kneeling during childhood, increasing the risk of knee pain associated with patella alta among children with CP [15]. Among the forms of spastic diplegia, children have been described as having a sensory function disorder resulting from difficulty in collecting information required for motor control, leading to inability to produce the necessary specific movements. This can be observed, for example, in children who are capable of walking with support but require facilitation by a caregiver walking near or behind them when not using an assisting device [35]. There are, to date, no valid instruments to objectively measure perceptual disorder with respect to motor function, even if perceptual disturbances are considered a constituent in CP diagnosis [1]. Clinical signs aimed to distinguish the perceptual disorder influencing movement in children with bilateral spastic CP have been reported, of which exaggerated startle reaction of the upper limbs showed the highest intra-observer reliability [36,37]. When retrospectively verifying the presence of the same perceptual signs in 32 children with diplegic CP aged 1–8 years, the above-mentioned signs as well as posture freezing were the easiest signs to recognize with the highest agreement [37]. These suggested clinical signs must be further evaluated and tested for reliability [36,37].

To improve mobility in children and young people with CP at GMFCS levels I–IV, mobility training using a goal-directed method is recommended across all motor subtypes. To improve walking velocity and endurance, both overground training and treadmill training has been suggested for GMFCS III [4]. In a recent study, the probability of children and adults classified in GMFCS levels III to be able to walk without support was estimated as doubtful. Given the dependency on others for mobility in the community in these children, increased access to independent, wheeled mobility is emphasized [38]. Expanding the understanding of a child’s motor behavior by adding a sensorimotor perspective when assessing a child’s motor function and gait could contribute to relevant choices when selecting interventions for utmost independence and mobility training in children at various GMFCS levels. For the purpose of differentiating children with pronounced clinical signs of perceptual disorder from those more influenced by motor difficulties, the clinical signs of perceptual disorder can be useful [36,37].

This study has limitations that need to be acknowledged. Crouch gait in CP often involves the consequence of skeletal malalignment at the hip, knee, and foot [5]. Not having presented these measurements in the present study could therefore have been a possible source of bias. Another limitation is the small number of participants, particularly at GMFCS level I; a larger study sample would strengthen the results. Nonetheless, discriminating between the passive knee extension at the physical assessment and maximum knee extension while weight bearing could contribute to further understanding of flexed knee gait and its causes in ambulating children with spastic bilateral CP.

## 5. Conclusions

This paper highlights the difference between knee position during walking versus knee contractures in children with bilateral spastic CP. In this study group, knee flexion during walking was found to be significantly greater than knee contractures in children at GMFCS levels II and III but not at GMFCS level I. During walking, the possible range of knee extension as measured in the physical assessment was utilized in children at GMFCS level I, whereas the children at GMFCS II and III levels did not utilize their available knee extension range. To complete the TUG test, increasingly more time was taken with greater knee flexion angles, indicating that motor function may be influenced by both motor and sensory aspects. The findings of this study point to the necessity of taking into account the sensory part of the sensorimotor relationship when assessing movement in children with bilateral spastic CP.

## Figures and Tables

**Table 1:** Distribution of age, weight, height, joint contractures, and orthopedic surgeries performed in children with bilateral spastic cerebral palsy (BSCP) gross motor function classification system (GMFCS) levels I to III and in children with typical development (TD). An asterisk indicates statistical significance (*p* < 0.05).

	TD (*n* = 22)	GMFCS I (*n* = 5)	GMFCS II (*n* = 12)	GMFCS III (*n* = 13)	*p* Value
Age median (min, max), years	8.9 (6.5, 16.9)	10.3 (8.6, 15.9)	11.8 (8.2, 15.8)	10.6 (7.6, 17.1)	0.635
Weight median (min, max), kg	33.3 (22.3, 81.8)	46.3 (31.6, 52.7)	40.2 (26.4, 99.7)	29.4 (18.5, 56.2)	0.601
Height median (min, max), cm	140 (121, 181)	145 (132, 164)	153 (130, 166)	137 (110, 170)	0.385
Joint contracture < neutral position in support limb expressed as “0”, median (min–max) degrees					
Ankle plantarflexion	0	0	0 (0, 5)	0 (0, 5)	0.530
Knee flexion	0	0	0 (0, 15)	0 (0, 25)	<0.001 *
Hip flexion	0	0	0 (0, 15)	0 (0, 15)	0.167
Orthopedic surgery performed (number of children)	-	1	7	2	0.065
Strayer (number of surgeries in both limbs)	-	1	3	0	0.175
Achilles tendon lengthening (number of surgeries in both limbs)		1	5	2	0.323
Medial hamstrings (number of surgeries in both limbs)		0	1	0	0.472

**Table 2:** Knee joint angles during walking at initial contact (KneeAngleIC) and at minimum knee flexion in stance (MinKneeFlexSt), presented as median (min, max) values in degrees, and time to accomplish the Timed Up and Go test (TUG), presented as median (min, max) values in seconds (s), in children with bilateral spastic cerebral palsy, GMFCS levels I to III, and in children with typical development (TD). Plus (+) indicates knee flexion, (−) indicates knee extension. An asterisk indicates statistical significance (*p* < 0.05).

	TD *n* = 22	GMFCS I *n* = 5	GMFCS II *n* = 12	GMFCS III *n* = 13	*p* Value
KneeAngleIC (°) median (min, max)	4.2 (−2.0, 12.0)	15.9 (2.5, 23.5)	27.1 (17.9, 68.7)	45.3 (10.7, 71.9)	<0.001 *
MinKneeFlexSt (°) median (min, max)	2.5 (−6.2, 9.0)	1.2 (−0.1, 14.3)	19.2 (0.8, 68.7)	37 (−3, 63.1)	<0.001 *
TUG (s) median (min, max)	8.2 (6.5, 10.6)	8.1 (7.4, 10.3)	12.3 (9.5, 17.1)	25.6 (11, 70)	<0.001 *

## Data Availability

The datasets generated and analyzed during the current study are available from the corresponding author on reasonable request. The data are not publicly available due to privacy and ethical restrictions.
